# CEACAM3—A Prim(at)e Invention for Opsonin-Independent Phagocytosis of Bacteria

**DOI:** 10.3389/fimmu.2019.03160

**Published:** 2020-02-11

**Authors:** Patrizia Bonsignore, Johannes W. P. Kuiper, Jonas Adrian, Griseldis Goob, Christof R. Hauck

**Affiliations:** ^1^Lehrstuhl Zellbiologie, Fachbereich Biologie, Universität Konstanz, Konstanz, Germany; ^2^Konstanz Research School Chemical Biology, Universität Konstanz, Konstanz, Germany

**Keywords:** phagocytosis, CEACAM3, pathogenic bacteria, granulocyte, human innate immunity, ITAM, immunoreceptor tyrosine-based activation motif, signal transduction

## Abstract

Phagocytosis is one of the key innate defense mechanisms executed by specialized cells in multicellular animals. Recent evidence suggests that a particular phagocytic receptor expressed by human polymorphonuclear granulocytes, the carcinoembryonic antigen-related cell adhesion molecule 3 (CEACAM3), is one of the fastest-evolving human proteins. In this focused review, we will try to resolve the conundrum why a conserved process such as phagocytosis is conducted by a rapidly changing receptor. Therefore, we will first summarize the biochemical and structural details of this immunoglobulin-related glycoprotein in the context of the human CEACAM family. The function of CEACAM3 for the efficient, opsonin-independent detection and phagocytosis of highly specialized, host-restricted bacteria will be further elaborated. Taking into account the decisive role of CEACAM3 in the interaction with pathogenic bacteria, we will discuss the evolutionary trajectory of the CEACAM3 gene within the primate lineage and highlight the consequences of CEACAM3 polymorphisms in human populations. From a synopsis of these studies, CEACAM3 emerges as an important component of human innate immunity and a prominent example of a dedicated receptor for professional phagocytosis.

## Introduction

The ability to detect and phagocytose microbes is vital to protect multicellular organisms against dangerous infections. In mammals, this important function is accomplished by dedicated immune cells, the so-called professional phagocytes, encompassing macrophages, dendritic cells, and polymorphonuclear granulocytes (PMNs). They carry out phagocytosis via two distinct mechanisms: On the one hand, they perform opsonin-independent phagocytosis by utilizing receptors such as mannose receptor, scavenger receptor, Siglecs, DC-SIGN, or Dectin-1, which directly recognize and bind microbial surfaces that expose characteristic molecular patterns, such as glycan structures with terminal mannose or sialic acid residues, or fungal β-glucans (1–3). As such types of glycans are found on various microorganisms, including bacteria, fungi, as well as protozoa, and can also occur on endogenous structures, opsonin-independent receptors often detect a broad and diverse range of particles. On the other hand, professional phagocytes are capable of performing opsonin-dependent phagocytosis. Prominent opsonin-dependent receptors are complement or Fc receptors, which require prior coating of particles with host-derived complement components or specific antibodies before they are able to initiate phagocytosis (4–6). Therefore, opsonin-dependent receptors can be targeted toward specific microbes, but they cannot support phagocytosis in situations where opsonins are either not present or where they fail to mark the microbial surface, for example, in the case of antigenically variable or encapsulated microorganisms.

**Figure 1:**
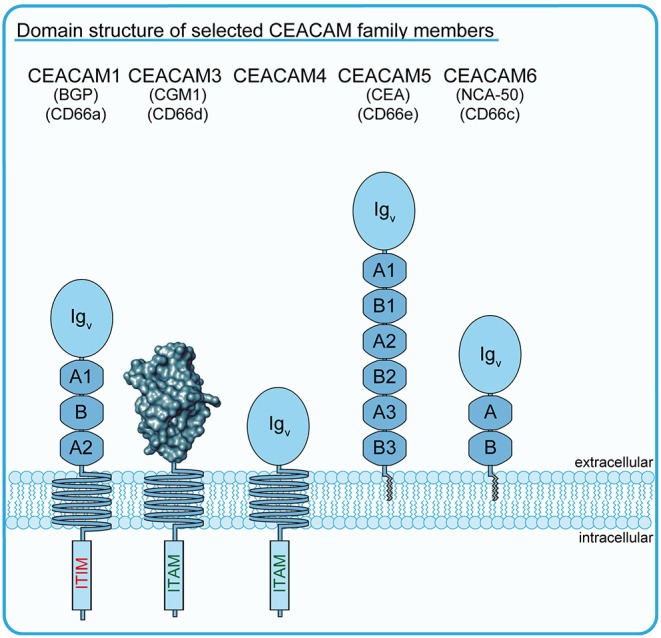
Schematic drawing of selected members of the human CEACAM family. Schematic outline of several members of the human carcinoembryonic antigen (CEA)-related cell adhesion molecule (CEACAM) receptor family. All CEA-related proteins belong to the immunoglobulin (Ig) superfamily and are characterized by the possession of a homologous amino-terminal Ig variable (Ig_V_)-like domain, which is depicted in the case of CEACAM3 as a rendered protein surface according to Bonsor et al. (7). The blue circles indicate the Ig_V_-like domains of CEACAMs other than CEACAM3, while the blue octagons indicate additional Ig constant 2 (Ig_C2_)-like domains occurring in different numbers in particular family members. The transmembrane helices of CEACAM1, CEACAM3, and CEACAM4 connect the extracellular Ig-domains with functional ITIM (CEACAM1), ITAM-like (CEACAM3), or consensus ITAM sequences (CEACAM4). GPI-anchors of CEACAM5/CEA and CEACAM6 are depicted in gray.

Recent work has indicated that, at least in primates, a third group of specialized phagocytic receptors operates, which combines pathogen-specific detection with the immediate action of opsonin-independent receptors. The paradigm for this type of phagocytic receptors is the carcinoembryonic antigen-related cell adhesion molecule 3 (CEACAM3). CEACAM3 is a receptor of the immunoglobulin (Ig) superfamily and a member of the CEA subfamily of Ig domain containing cell adhesion molecules (IgCAMs) (Figure 1). In humans, CEACAM3 is selectively expressed by PMNs and plays a prominent role in the opsonin-independent detection and elimination of a small set of human-restricted bacteria. In this review, we will place CEACAM3 in the context of a growing list of bacterial pathogens expressing CEACAM-binding adhesins and discuss the biochemical and functional evidence that this receptor is an effective phagocytosis-initiating protein and granulocyte activator. Further, we will elaborate the evolutionary trajectory of the CEACAM3 gene within the primate lineage and discuss the significance of human CEACAM3 polymorphisms, which appear to accommodate the recognition of variable bacterial surface antigens.

## CEACAM Family Members and Their Role as Microbial Targets

Upon the identification of carcinoembryonic antigen (CEA) as a prominent surface protein expressed by human colon carcinomas, it was soon realized that antibodies directed against CEA react with numerous other proteins, especially on granulocytes (8, 9). According to their apparent molecular weights, these proteins were initially termed non-specific cross-reacting antigen (NCA) -26, -50, -90, -95, and -160 (10). Screening of a human leukocyte cDNA library with a probe derived from NCA-50 uncovered several transcripts including clone W264 containing a 1259-base pair insert (11). The insert encoded a 252-amino acid protein, which was designated Carcinoembryonic Gene family Member 1a (CGM1a) and later grouped, due to its reactivity with monoclonal antibodies, into the CD66 cluster of differentiation. Besides CGM1a (CD66d), the CD66 antigens comprise biliary glycoprotein (BGP; CD66a), CGM6 (CD66b), NCA-50 (CD66c), and CEA (CD66e), which share 69–92% amino acid sequence identity in their amino-terminal immunoglobulin-variable (Ig_V_)-like domains with CGM1a (11–14). Superposition of known crystal structures of CEA, CD66a, and CD66c reveals that these sequence similarities also translate into high structural conservation between these proteins (7, 15).

The growing awareness of the complexity of the CEA family necessitated a major revision of the nomenclature, which led to CGM1a (CD66d) being renamed CEACAM3 (16) (for current and former nomenclature of CEACAM family members discussed in this review, please consult Figure 1). While CEACAM3 transcripts and protein have only been detected in human granulocytes and myeloid leukemia cells, the other closely related CD66 antigens are either widely expressed on epithelial and hematopoietic cells (CD66a/BGP/CEACAM1 as well as CD66c/NCA-50/CEACAM6) or exclusively expressed by mucosal epithelial cells (CD66e/CEA/CEACAM5) (17–19). Furthermore, CEACAM3 is distinct from other CD66 antigens in that its extracellular part does only comprise a single Ig_V_-like domain and lacks additional Ig constant (Ig_C_)-like domains, which are present in varying numbers (2-6 Ig_C_-like domains) in CEACAM1, CEACAM5, and CEACAM6 (Figure 1) (20). This short stature of CEACAM3 might also be the reason why this receptor does not participate in binding interactions with other CEACAM family members, as the Ig_C_-like domains can stabilize cis- and trans-interactions between CEACAM extracellular domains and thereby contribute to cell–cell adhesion (21, 22). Indeed, CEACAM1, CEA, CEACAM6, and CEACAM8 engage in CEACAM–CEACAM interactions with each other to support cell–cell binding (12, 21–24).

As CEACAM3 does not participate in these binding interactions, what could then be the function of this particular CEA-related protein on professional phagocytes? Clearly, the physiological role of CEACAM3 can only be reconciled in light of the fact that several pathogenic bacteria and fungi take advantage of epithelial CEACAMs as preferred docking sites on the mucosa [for review, see (25)]. Indeed, a growing list of pathogens has been found to express dedicated adhesins to specifically connect to human CEACAM family members, such as CEACAM1, CEA, and CEACAM6, which are exposed on the apical surface of human epithelial cells. CEACAM-binding microorganisms comprise *Neisseria gonorrhoeae* (causative agent of the venereal disease gonorrhea), *Neisseria meningitidis* (bacterial meningitis), *Haemophilus influenzae* (pneumonia, bacterial meningitis), *Haemophilus aegyptius* (purulent conjunctivitis), *Helicobacter pylori* (chronic gastritis, stomach cancer), *Moraxella catarrhalis* (otitis media, sinusitis), *Fusobacterium nucleatum* (periodontal disease), pathogenic *Escherichia coli* strains (Adherent-invasive *E. coli*, Diffusely adherent *E. coli*; involved in Crohn's disease), and the yeast *Candida albicans* (candidiasis, systemic infections) (26–35). It is important to mention that almost each of these pathogens employs a structurally distinct adhesive protein to bind human CEACAMs, implying that these adhesins have evolved independently multiple times in a striking form of convergent evolution (7, 15, 32, 36–39). Evidently, there must be strong, but not necessarily a uniform selection pressure on these microorganisms to develop CEACAM-binding adhesins. Several non-mutually exclusive explanations have been put forward to explain this exceptional preference of distinct pathogenic microbes to engage human CEACAMs. One finding relates to the fact that CEACAM1, the target of a large fraction of these adhesins, is also expressed by T cells and that major CEACAM1 isoforms have a negative regulatory role in T cell stimulation and proliferation [reviewed in (40)]. A second hypothesis is based on the fact that a unifying theme for all CEACAM-binding microbes is their outstanding ability to colonize, often throughout the lifespan of an individual, the mucosal surface of either the naso-pharynx, the gastrointestinal, or the urogenital tract. The role of CEACAM engagement in mucosal colonization has been best worked out in the case of *N. gonorrhoeae* and *N. meningitidis* and demonstrated that both microbes greatly profit from tight association with CEACAMs, which facilitates successful host colonization (41–43). Aside from their role as a handle by which to anchor to the mucosal epithelia, CEACAM engagement allows bacteria to suppress the exfoliation and delamination of superficial epithelial cells, thereby creating a stable foothold on the mucosa (41, 44, 45). It becomes obvious that pathogens can immensely profit, potentially in multiple ways, from engaging CEACAMs on epithelial cells and this nicely explains the prevalence and independent evolution of CEACAM-binding adhesins among human pathogens. However, why is it then that humans rarely succumb to gonococcal infection or develop severe forms of disease after being colonized by *N. meningitidis* or *H. pylori*, which are present in a large fraction of the healthy population? Indeed, CEACAM-binding pathogens such as *F. nucleatum, M. catarrhalis, N. gonorrhoeae*, or *H. pylori*, despite being able to efficiently colonize the human mucosa, rarely or only in a minority of the cases lead to a fatal outcome. It is exactly in the context of this scenario that we can now appreciate the role of CEACAM3, a CEACAM family member that does not participate in cell–cell interactions, but is present on the surface of professional phagocytes. In particular, the capacity of CEACAM3 to trigger rapid phagocytosis of attached particles and to activate bactericidal mechanisms of granulocytes will be discussed in the next sections, as these features provide major clues to understand the specialized function of this protein.

## CEACAM3-Initiated Signal Transduction Leading to Phagocytosis

**Figure 2:**
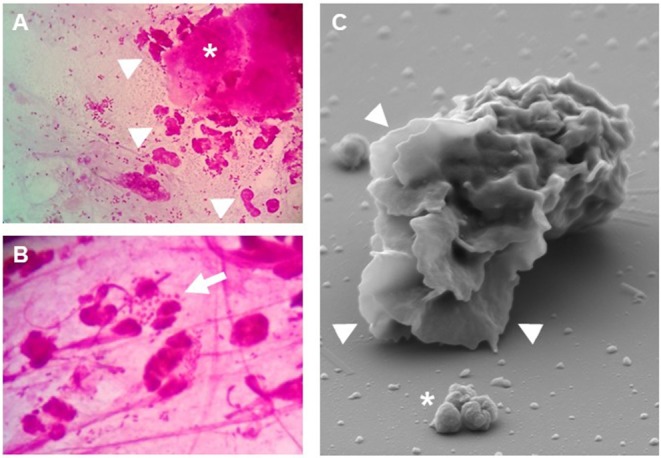
Granulocytes respond to *Neisseria gonorrhoeae*. **(A,B)** Gram stain of cervical smears from *N. gonorrhoeae*-infected women. Even at low magnification **(A)** the presence of granulocytes (arrowheads) and detached epithelial cells (asterisk) can be readily detected. **(B)** At higher magnification, granulocytes with phagocytosed diplococci (arrow) as well as extracellular bacteria are visible. **(C)** Scanning electron microscopy highlights the massive lamellipodia (arrowheads) induced on isolated primary human granulocytes upon exposure to CEACAM3-binding *N. gonorrhoeae* (asterisk).

CEACAM3's notable status within the CEACAM family is not only due to its small extracellular domain and its cell-type-specific expression pattern, but is also based on a particular sequence motif within its cytoplasmic domain. Similar to the prototypic opsonin-dependent phagocytic receptors of the Fc receptor family, the carboxy-terminus of CEACAM3 encompasses an immunoreceptor tyrosine-based activation motif (ITAM) [for review, see (46)]. To be more precise, the motif found in CEACAM3 does not conform perfectly to the consensus ITAM (*D/Ex*_(7)_*D/ExxYxxI/Lx*_(6−8)_*YxxI/L*), but resembles an ITAM-like motif, where the carboxy-terminal leucine/isoleucine residue is substituted by methionine (47–49). The presence of this motif and the expression in professional phagocytes already indicate that CEACAM3 might be involved in phagocytosis of CEACAM-binding bacteria. For several CEACAM-binding pathogens, granulocytes play a major role during symptomatic disease. For example, the purulent exudate containing numerous granulocytes with intracellular, gram-negative diplococci is a diagnostic hallmark of gonorrhea (Figures 2A,B). It has long been known that gonococci, which express a CEACAM3-binding adhesin, are recognized and phagocytosed by human granulocytes in an opsonin-independent manner (Figure 2C), while isogenic strains lacking a CEACAM-binding adhesin are hardly recognized under these conditions (26, 27, 50–52). Despite the presence of other CEACAM family members such as CEACAM1 and CEACAM6 on the granulocyte surface and despite the fact that CEACAM3 is expressed at lower levels compared to CEACAM1 and CEACAM6, CEACAM3 is the main driving force behind this rapid and efficient opsonin-independent phagocytosis (53). Evidence for the prominent role of CEACAM3 comes from pharmacological, biochemical, genetic, and microbiological approaches: inhibitors that affect CEACAM1 and CEACAM6-mediated uptake in transfected cell lines (such as cholesterol-depleting agents) do not interfere with the opsonin-independent phagocytosis of CEACAM-binding bacteria by granulocytes (54), while inhibitors or blocking antibodies selectively affecting CEACAM3-mediated uptake in transfected cell lines also disrupt this process in granulocytes (50). Interference with CEACAM3-specific binding partners or signaling processes by transduction of primary human granulocytes with dominant-negative variants also severely compromises the opsonin-independent uptake of CEACAM-binding bacteria. Selective expression of CEACAM3, but not CEACAM1 or CEACAM6, in murine promyelocytic cells can recapitulate major features of neutrophil activation in response to CEACAM-binding bacteria such as oxidative burst and degranulation (55). Furthermore, microbes expressing particular adhesins, which bind CEACAM1, but not CEACAM3, are hardly phagocytosed by primary human granulocytes in the absence of opsonins (56). Therefore, the immediate and dramatic phagocytic response of human granulocytes exposed to CEACAM-binding bacteria can be mainly attributed to CEACAM3.

A number of studies have addressed the molecular basis of CEACAM3's capability to vigorously trigger opsonin-independent phagocytosis. Most of these investigations, conducted with either transfected human cell lines or primary human granulocytes, have pointed toward a major role of the ITAM-like motif for CEACAM3 functionality in phagocytosis. For example, phosphorylation of the tyrosine residues within this motif (Y230/Y241) is critical for CEACAM3-initiated phagocytosis, as mutation of either tyrosine to a phenylalanine significantly decreases internalization and mutation of both residues results in an additive effect (47, 48, 53, 57). Interestingly, a single tyrosine-to-phenylalanine mutation completely blocked phosphorylation of CEACAM3 (48). Whether this points to a cooperative phosphorylation mechanism requiring both tyrosine residues or is due to inadequate sensitivity of the assay is unclear. Besides the ITAM-like motif, additional structural elements within the cytoplasmic domain possibly contribute to phagocytic signaling as the CEACAM3 Y230F/Y241F double mutant exhibits residual phagocytic activity compared to variants, which lack the complete cytosolic domain (48, 57). In contrast to CEACAM1 and CEACAM6, cholesterol-rich membrane domains (lipid rafts) do not seem to contribute to CEACAM3-mediated phagocytosis, as the CEACAM3-dependent internalization of bacteria is insensitive to severe cholesterol depletion, e.g., by methyl-β-cyclodextrin (54, 58, 59). It has been proposed that a Y-to-F mutation in the ITAM motif generates a binding site for AP-2, which could support an endocytic mode of internalization (48). However, regular endocytosis via AP-2 initiated, clathrin-coated vesicles has an upper size limit of 200 nm (60), implying alternative endocytic processes upon deletion or mutation of the CEACAM3 ITAM-like sequence. Though it is currently unknown which specific cellular processes guide the residual, ITAM-independent internalization of bacteria, the ITAM-dependent events upon CEACAM3 stimulation have been extensively analyzed.

**Figure 3:**
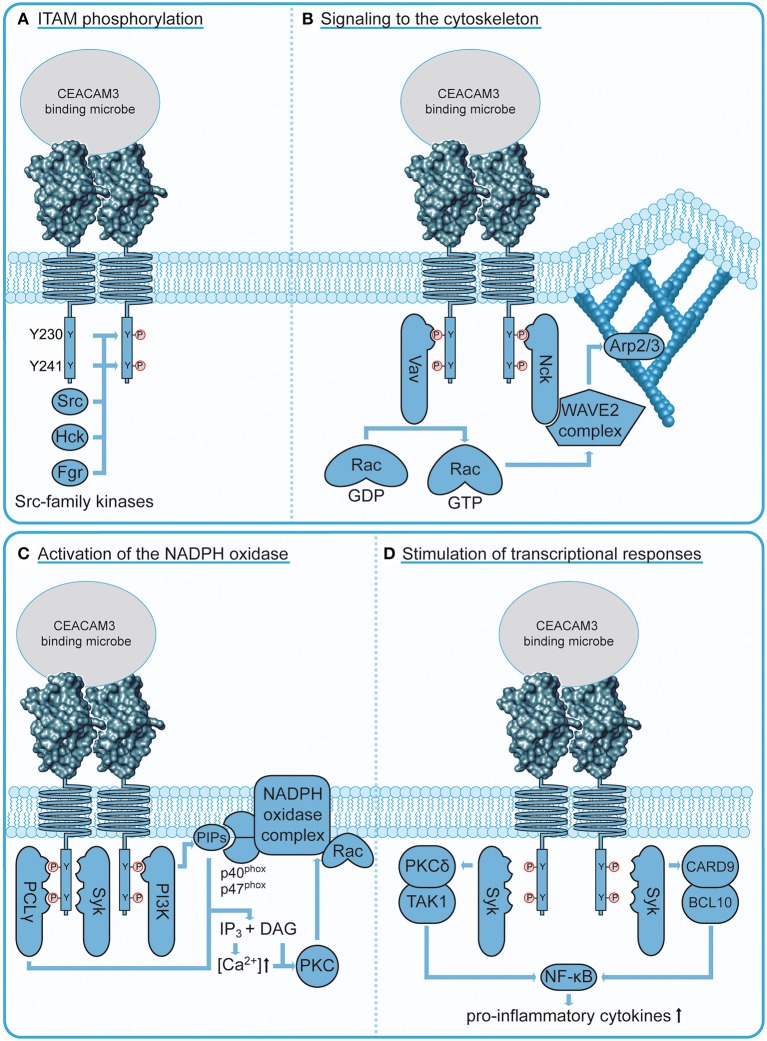
CEACAM3 signaling connections. **(A)** Phosphorylation of CEACAM3 upon bacterial engagement. Localized clustering of CEACAM3 receptors by multivalent, CEACAM-binding bacteria trigger recruitment and activation of several Src family tyrosine kinases, which in turn phosphorylate the ITAM-like sequence in the cytoplasmic domain of the receptor at two tyrosine residues (Y230 and Y241). This phosphorylation event is critical for downstream signaling processes. **(B)** CEACAM3 signaling to the cytoskeleton. The adaptor protein Nck binds via its SH2 domain to the membrane proximal CEACAM3 phosphotyrosine residue pY230 and recruits the WAVE2 complex to sites of bacterial attachment. Activation of this complex requires a localized increase in GTP-loaded Rac, which is orchestrated by recruitment of the Rac guanine nucleotide exchange factor Vav. Similar to Nck, Vav binds via its SH2 domain to CEACAM3 pY230. Activation of the WAVE2 complex leads to localized activation of Arp2/3 at the site of infection resulting in particle engulfment by actin cytoskeleton-based lamellipodial protrusions. **(C)** CEACAM3 signaling to the NADPH oxidase. Residue pY230 serves as an interaction platform for the regulatory subunit of phosphatidylinositol 3′ kinase (PI3K). PI3Ks are responsible for the generation of phosphoinositides (PIPs), which regulate the oxidative burst by recruitment of NADPH oxidase subunits p40^phox^ and p47^phox^. In addition, PIP hydrolysis by CEACAM3-recruited phospholipase C γ (PLCγ) releases the second messengers inositol-(1,4,5)-trisphosphate (IP_3_) and diacyl glycerol (DAG). Together, they activate the protein kinase C (PKC), which further stimulates the NADPH oxidase complex. **(D)** CEACAM3 stimulation of neutrophil transcriptional response. CEACAM3 can trigger the NF-κB-mediated transcriptional regulation of IL-8 and other neutrophil chemotactic factors. CEACAM3-initiated NF-κB activation occurs via two distinct routes: the PKCδ/TAK1 pathway and the CARD9/BCL10 pathway depend on Syk localization to phosphorylated CEACAM3.

Genetic, biochemical, and pharmacological evidence supports a major role for kinases of the Src family in CEACAM3 phosphorylation (Figure 3A). Indeed, the local clustering of CEACAM3 by the multivalent bacteria triggers recruitment and activation of several members of the Src family tyrosine kinases, including Hck and Fgr in granulocytes, while in transfected cell lines, Src, Yes, and Fyn might take over the respective role (48, 52, 61, 62). Due to acyl modification, Src family kinases are constitutively associated with the cytoplasmic leaflet of membranes and are therefore in a prime position to initially phosphorylate the ITAM tyrosine residues. Although the tyrosine kinase Syk is also recruited to nascent gonococci-containing phagosomes in an ITAM-dependent fashion, pharmacological inhibition of Syk did not reduce bacterial internalization (63). Only when polystyrol beads larger than 5 μm (a typical gonococcal diplococcus is about 1–2 μm in size) were coated with anti-CEACAM IgG and used as bacterial surrogate did Syk augment internalization. The fact that Syk facilitates phagocytosis depending on particle size is not unique to CEACAM3 as the same phenomenon has been observed for FcγR-mediated phagocytosis (64). Therefore, Syk is dispensable for internalization of gonococci, but it clearly does promote downstream bactericidal activity by enhancing the oxidative burst, degranulation, and the NF-κB-mediated inflammatory response (63).

Upon phosphorylation, the ITAM-like motif in CEACAM3 creates a platform for effectors that drive cytoskeletal remodeling required for phagocytic cup formation (Figure 3B). The adaptor molecule Nck binds CEACAM3 in a phosphorylation-dependent manner via its SH2 domain and recruits the WAVE2 complex to sites of bacterial attachment (65). WAVE2 is part of a multiprotein complex that activates Arp2/3-mediated actin nucleation, which drives lamellipodia extension [reviewed in (66)]. Indeed, ablation of Nck or inhibition of WAVE2 impedes lamellipodia formation and bacterial uptake (65). The WAVE2 complex is a coincidence detector, which is activated by the local and temporal co-occurrence of protein tyrosine phosphorylation, acidic phospholipids, and activation of the small GTPase Rac (67). Importantly, all of these events are concomitantly initiated by CEACAM3 engagement. Acidic phospholipids such as phosphatidylinositol-(3,4,5)-trisphosphate (PIP3) and phosphatidylinositol-(3)-phosphate (PI3P) sequentially accumulate at the phagocytic cup during engulfment (59, 68, 69). WAVE2 activation strictly depends on the recruitment of the small Rho GTPase, Rac, which is crucial for remodeling of the cytoskeleton (70). Rac1 is also essential for CEACAM3-mediated phagocytosis and is locally and transiently activated at the phagocytic cup (52, 53, 57). Activation of Rac is catalyzed by specific guanine nucleotide exchange factors (GEFs) that exchange Rac1-bound GDP for GTP and CEACAM3 aggregation triggers Rac GTP loading by the GEF Vav (61). Interestingly, Vav itself is activated by Src family kinases (71, 72) and the Vav SH2 domain can interact directly with the phosphorylated Y230 within the ITAM-like motif of CEACAM3 (61). As most other GEFs bind via their pleckstrin homology domain (PH domain) to phosphoinositides, their recruitment is intrinsically dependent on PI3K activity. In contrast, Vav's direct interaction with phosphorylated CEACAM3 could render this process PI3K-independent. Indeed, phosphoinositide 3′ kinase (PI3K) activity is dispensable for CEACAM3-mediated internalization of gonococci (68, 73). The direct interaction between phosphorylated CEACAM3 and a Rac GEF as well as the direct linkage to the WAVE complex via Nck could explain why CEACAM3-mediated phagocytosis is particularly efficient and rapid (69). *In vitro*, ~90% of primary human granulocytes have internalized multiple CEACAM-binding gonococci within 20 min of infection, while CEACAM-non-binding gonococci remain almost untouched (48, 69). These studies of CEACAM3-initiated signal transduction have also indicated that the ability of this receptor to trigger phagocytosis relies on cellular constituents, such as Src family kinases, Vav, Rac, and Nck-WAVE, which can be found in almost any mammalian cell type. Maybe this universality of CEACAM3 downstream connections explains why transfection of CEACAM3 cDNA in diverse cell types, from cervical epithelial cells to mouse fibroblasts, is sufficient to convert such non-professional phagocytes into cells avidly and efficiently internalizing CEACAM-binding bacteria. The generic nature of these processes also indicates that studies of CEACAM3-initiated phagocytosis are helpful to delineate the core elements necessary for productive internalization of particles.

## CEACAM3-Initiated Signal Transduction Beyond Phagocytosis

In professional phagocytes, phagocytosis is tightly coupled to downstream bactericidal processes. One central regulatory switch in this regard is the GTPase Rac, which not only regulates cytoskeletal remodeling, but is also an essential subunit of the NADPH oxidase, the enzyme complex generating the microbiocidal oxidative burst (Figure 3C). In contrast to the PI3K-independent Rac activation during engulfment described above, the CEACAM3-induced oxidative burst strictly depends on PI3K activity (73). Since there is only a partial temporal overlap between particle engulfment and the oxidative burst, it is possible that Vav-mediated GTP-loading of Rac during engulfment is disconnected from a putative phosphoinositide-dependent GEF, which might activate Rac later during the induction of an oxidative burst. However, PI3K-generated phosphoinositides are required at additional regulatory steps during NADPH oxidase assembly, such as the recruitment of NADPH oxidase subunits p40^phox^ and p47^phox^ through their PI3P-binding PX domains (74). Phosphoinositides also serve as substrate for various lipid phosphatases and phospholipases. Interestingly, the SH2 domain of phospholipase C γ (PLCγ) can bind CEACAM3 *in vitro* and the isolated SH2 domain is enriched around gonococci-containing phagosomes (48, 75). PLCγ-mediated hydrolysis of phosphatidylinositol-(4,5)-bisphosphate (PIP2) produces the second messengers diacyl glycerol (DAG) and inositol-(1,4,5)-trisphosphate (IP_3_), which in turn trigger increases in cytosolic Ca^2+^ and PKC activation. Indeed, intracellular Ca^2+^ levels in PMNs rapidly rise upon CEACAM3 engagement and this process does not occur in cells lacking PLCγ (47). Accordingly, upstream PLCγ activity might be required to allow PKC-mediated phosphorylation of multiple NADPH oxidase subunits, which represents an important regulatory step in activation of the oxidative burst [reviewed in (76)].

Though both tyrosine residues within the ITAM-like sequence of CEACAM3 seem to be functionally relevant, the biochemical assays conducted so far have pointed toward the Y230 residue as the central hub for interactions with SH2 domain-containing proteins. This conclusion is based on binding studies with synthetic phospho-peptides and pull-down experiments with recombinant proteins, which demonstrate that the SH2 domains of Src family kinases, PI3K, Nck, or Vav selectively bind to phospho-Y230. Therefore, the CEACAM3 ITAM-like sequence has been likened to a so-called HemITAM sequence found, for example, in the macrophage receptor Dectin-1, where also a single tyrosine residue conveys the phagocytic function (77, 78). Interestingly, the only known negative regulator of CEACAM3-mediated signaling, the adaptor protein Grb14, also targets phospho-Y230 (79). One can easily envision that Grb14 restricts access for other SH2 domain-containing effector proteins and thereby interferes with CEACAM3-mediated phagocytosis. However, the idea that multiple proteins compete for phospho-Y230 of CEACAM3 immediately begs the question how these binding events can be coordinated to allow a productive and orchestrated cellular response. In the future, time-resolved analysis of the various SH2 domain-mediated binding events upon bacterial CEACAM3 engagement might help to answer this question. Nevertheless, the emerging overall picture of CEACAM3 phosphorylation-initiated events depicts Y230 within the ITAM-like motif as the minimal structural feature, which directly links CEACAM3 engagement with cytoskeletal remodeling as well as with the initiation of bactericidal responses.

It is interesting to mention that there is a further member of the CEACAM family, CEACAM4, which has a domain architecture similar to CEACAM3, which is selectively expressed in granulocytes, and which harbors a consensus ITAM (*D/ExxYxxLx*_(6−8)_*YxxI*) (Figure 1). CEACAM4 is an orphan receptor, as neither an endogenous nor a microbial ligand for this membrane protein has been detected. However, the cytoplasmic domain of CEACAM4 can trigger particle uptake, with both ITAM-embedded tyrosine residues engaging in SH2 domain interactions (80). Therefore, the human CEACAM family appears to harbor additional phagocytic receptors that might function to eliminate microorganisms in an opsonin-independent manner.

Although the main task of PMNs is to clear pathogens through their bactericidal capabilities, there is mounting evidence that they can also shape the inflammatory response [reviewed in (81, 82)]. Indeed, CEACAM3 activation can trigger an inflammatory program in PMNs (Figure 3D). CEACAM3 binding by *Moxarella catarrhalis* activates the CARD9/BCL10 pathway resulting in NF-κB-mediated expression of the potent neutrophil chemotactic factor, interleukin-8 (IL-8) (83). As CEACAM3 expression is restricted to human neutrophils, its contribution to inflammatory responses in an organismal context are difficult to study. Sintsova et al. made use of a transgenic mouse model that harbors a 187-kb human bacterial artificial chromosome encoding CEACAM3, CEACAM5, CEACAM6, and CEACAM7 (CEABAC mice) (84) to study the CEACAM-mediated response to infection with *N. gonorrhoeae* (85). Global expression profiles were generated from both WT and transgenic neutrophils infected with CEACAM-binding gonococci to discern CEACAM-dependent signatures. A pronounced upregulation of pro-inflammatory cytokines was observed in neutrophils from CEABAC mice, which depends on p38 MAPK activity and the PKCδ/TAK1/NF-κB axis (85). Though the used transgenic neutrophils and primary human granulocytes express other CEACAMs, which could be engaged by CEACAM-binding bacteria (such as CEACAM6 in the case of transgenic murine neutrophils or CEACAM1 and CEACAM6 in the case of primary human neutrophils), inhibitor studies point to CEACAM3-ITAM signaling as the main contributor to neutrophil-initiated inflammatory responses. *In vivo*, infection of CEACAM-transgenic, but not wild-type mice with *N. gonorrhoeae* led to increased neutrophil infiltration and increased levels of neutrophil-derived IL-1β and MIP-1α. Accordingly, CEACAM3 signaling on the one hand helps to limit gonococcal survival, but also initiates a vicious cycle, where the bacteria-triggered release of chemotactic cytokines leads to increased neutrophil influx and potentiates the risk of severe damage to the infected tissue (86). In this light, it will be important to understand how CEACAM3 signaling is kept in check to prevent unrestrained inflammatory signaling. In contrast to the initiation of CEACAM3 signaling, surprisingly little is known about its termination. The negative regulatory role of the adaptor protein Grb14 (79) has already been discussed above. Furthermore, phosphorylation of CEACAM3 appears to be counteracted by the cytoplasmic protein tyrosine phosphatase SHP-1, which most likely constrains CEACAM3 effector functions by compromising ITAM functionality (87). Interestingly, neutrophils also express CEACAM1, which contains an immunoreceptor tyrosine-based inhibitory motif (ITIM). However, co-recruitment of CEACAM1 does not seem to have an inhibitory effect on CEACAM3 phagocytic activity (55). Further research is required to address mechanisms that could regulate CEACAM3 activity, including intracellular trafficking of CEACAM3 and cooperation with other phagocytic receptors (e.g., Fc-γ and complement receptors). Considering the potential detrimental effects of excessive CEACAM3 activation, it is highly likely that in human PMNs, additional negative modulators of CEACAM3 signaling operate.

## CEACAM3 Evolution—a Red Queen Scenario at Work

Based on the functional studies summarized in the previous sections, it is safe to conclude that CEACAM3 represents an effective detector and eliminator of CEACAM-binding bacteria. In its capacity as a phagocytosis-promoting receptor, CEACAM3 can take care of pathogens expressing CEACAM-binding adhesins designed to exploit the human receptors for mucosal colonization. Thereby, CEACAM3 might help to establish a truce between host and pathogen, which could be one of the reasons why CEACAM-binding pathogens are contained in most instances. Looking at this reality from the viewpoint of a microbe, wouldn't it then be smart to avoid expressing a CEACAM3-binding adhesin in the first place? Obviously, bacteria unanimously answered this question with “Yes,” as they appear to optimize their adhesins to allow binding to epithelial CEACAMs such as CEACAM1 and CEA, while evading CEACAM3 recognition. Indeed, most CEACAM-binding bacterial adhesins analyzed in this regard show selective binding to either CEACAM1 or CEA. This is true for the CEACAM-binding Opa protein adhesins of *N. gonorrhoeae* and *N. meningitidis* as well as for their functional homolog, the OMP P1 adhesin of *H. influenzae*, which have been studied most thoroughly in this context. For example, a single gonococcal strain encodes 11 distinct Opa adhesins and the complete compendium of Opa proteins has been functionally tested for binding to CEACAM family members in the case of *N. gonorrhoeae* strains MS11 and VP1. In MS11, 10 out of 11 Opa adhesins bind CEA, while only three of these are also able to be recognized by CEACAM3 (50, 51, 88). For strain VP1, 8 out of 10 tested Opa adhesins bound to CEA or CEACAM1, while only a single Opa protein associated with CEACAM3 (56). In a complementary approach, Sintsova et al. screened a large collection of primary isolates from gonorrhea patients for their CEACAM-binding capacity. Instead of trying to analyze the complete Opa repertoire of each of these strains, the authors used low passage isolates, which are thought to continue to express *in vitro* the Opa variant previously selected for and expressed *in vivo* (89). Also in this study, the overwhelming majority with 74% or 80% of the strains bound CEA or CEACAM1, respectively, and only 27% were found to also recognize CEACAM3 (89). While most strains bound CEA and/or CEACAM1, but not CEACAM3, not a single strain could be observed, which showed a reverse binding pattern: recognizing CEACAM3, but not recognizing an epithelial CEA family member (89). An even more skewed situation is found in *N. meningitidis*, where 11 of 13 Opa proteins of serogroup A, B, and C strains, which have been tested in binding assays with different CEACAMs, associated with either CEA or CEACAM1, but none of those Opa proteins associated with CEACAM3 (90, 91). Similarly, all of the OMP P1 adhesins derived from 13 different strains of *H. influenzae* bound to CEACAM1, but none of those adhesins was recognized by CEACAM3 (32). Accordingly, bacteria seem to optimize their adhesins to discriminate between these closely related CEACAMs and to avoid recognition by CEACAM3.

If there is such a directed evolution on the side of bacterial CEACAM-binding adhesins, is there a discernable adaptation on the host side? The increasing availability of human and other primate genomic information has now allowed an unraveling of the evolutionary context of CEACAM3. It has been recognized before that homologs of several human CEACAMs are absent from rodents. More specifically, a CEACAM3 ortholog could not be identified even in Old World primates, such as baboon or African green monkey, by homology searches based on the extracellular Ig_V_-like domain (92). Large-scale genome comparisons between closely related primate species have revealed a high degree of non-synonymous vs. synonymous nucleotide changes in ortholog genes of CEACAM family members (93, 94). Interestingly, the bacterial receptors within the CEACAM family (CEACAM1, CEACAM3, CEA, and CEACAM6) show an exceptionally strong signature of positive selection, suggesting that they belong to the fastest-evolving human genes. In particular, the *CEACAM3* gene appears to be a recent evolutionary invention. Though ITAM-sequence containing CEACAM-related genes have been described for various mammals (95–97), a *CEACAM3* gene with its specific exon/intron structure and the characteristic ITAM-like sequence seems to occur only after the emergence of Old World monkeys (around 35 million years ago) (98). Indeed, a *CEACAM3* ortholog has only been detected at the syntenic locus in the genomes of baboon, macaque, orangutan, gorilla, chimpanzee, and humans (94). Due to uncertainties in the assembly of some genomes such as tarsier, lemurs, and lories, gene synteny is not a valid criterion to rule out the existence of CEACAM3 orthologs in lower primates. However, CEACAM3 orthologs share several discriminative features such as protein length or the presence of the characteristic HemITAM, which does not seem to occur in any lower primate genome supporting the idea that CEACAM3 emerged late in primate evolution. Despite the absence of a *CEACAM3* gene, ITAM-containing CEACAM transcripts are present in lower primates, such as transcript XR_001153184.2 in the mouse lemur. The ITAM encoded by XR_001153184.2 perfectly matches the ITAM consensus (YxxL−7AS–YxxI) as well as the sequence found in human CEACAM4 (80). Therefore, this transcript could originate from an ancestral primate CEACAM4 ortholog. It is plausible that CEACAM3 is the result of gene duplication and recombination between an ancestral *CEACAM1* gene (providing the Ig_V_ domain encoding exon) and an ancestral lower primate *CEACAM4* gene (providing the transmembrane and the intracellular domain encoding exons) (99). Indeed, the 3′ end of the large intron 2 of the *CEACAM3* gene bears similarities to intron 2 of the *CEACAM4* gene, supporting the idea that such a recombination event occurred at the advent of higher primates (99). Together, the genomic evidence suggests that CEACAM3, the specialized phagocytic receptor for CEACAM-binding bacteria, emerged relatively late during primate evolution, expanding the human innate immunity arsenal.

An interesting corollary of this continuing bacteria–host co-evolution can be seen by the occurrence of CEACAM3 polymorphisms in the human population. In fact, while people outside of Africa mainly express the common CEACAM3 allele, particular ethnic groups harbor CEACAM3 variants. For example, in several groups with African ancestry, a third of the population expresses a distinct CEACAM3 allele (minor CEACAM3 allele) (94). This minor allele carries four non-synonymous single-nucleotide changes, which convert the CEACAM3-Ig_V_ domain amino acid sequence into a near replica of the CEACAM1 Ig_V_ domain. Modeling of the polymorphic CEACAM3 Ig_V_ domain according to the known CEACAM3 crystal structure (7) reveals that these residues are surface exposed and three of the modified amino acids contribute to the CEACAM3 binding interface for various bacterial adhesins (94). It comes as no surprise that this minor CEACAM3 allele, which now mimics CEACAM1, shows a striking gain of function in that it recognizes additional bacterial adhesins, such as the OMP P1 protein of *H. influenzae*, which escape detection by the common CEACAM3 allele (94).

These recent studies have shed light on an ongoing arms race between several host-restricted CEACAM-binding pathogens and the human immune system. In a type of Red-Queen scenario, the diverse CEACAM-binding pathogens need to optimize their adhesins to escape CEACAM3 detection, while still retaining their ability to bind mucosal CEACAMs. As a consequence, human CEACAM3 rapidly evolves to keep up its function. Viewed from the angle of bacterial adhesin binding to distinct CEACAMs as well as from the perspective of human genomic variation, the phagocytic receptor CEACAM3 appears as a central element in the innate defense against this group of extremely specialized bacteria. However, one has to acknowledge that CEACAM binding preferences of bacterial adhesins as well as analyses of CEACAM3 evolution only provide circumstantial evidence for the importance of this innate immune receptor. Accordingly, a more direct test of CEACAM3 function in the context of innate immune responses within the intact organism would be desirable. Unfortunately, the standard genetic approach of employing knock-out animals, such as a “CEACAM3-knock-out” mouse, is not an option in this case. Moreover, CEACAM3-transgenic mice with granulocyte-selective expression of CEACAM3, in the absence of other CEACAM family members, do not exist currently.

In view of the increasing availability of human genomic information and corresponding medical records, it does not appear unrealistic to foresee a future, where novel insight into CEACAM3 function might come directly from combined genomic–phenotypic studies in humans (100). Indeed, the occurrence of an unexpected high proportion of loss-of-function alleles in particular human populations (101) could help to reveal plausible connections between CEACAM3 deficiency and susceptibility for particular infectious diseases. Despite the inherent limitations of research on a primate-only protein, the study of the phagocytic receptor CEACAM3 will surely continue to unearth fascinating aspects of human biology and of our constant and challenging interplay with the microbial world.

## Author Contributions

All authors listed have made a substantial, direct and intellectual contribution to the work, and approved it for publication.

### Conflict of Interest

The authors declare that the research was conducted in the absence of any commercial or financial relationships that could be construed as a potential conflict of interest.

## Abbreviations

CEA: carcinoembryonic antigen
CEACAM: CEA-related cell adhesion molecule
Ig: immunoglobulin
ITAM: immunoreceptor tyrosine-based activation motif.
